# Mentoring the Next Generation of Integrated Care Stakeholders: Lessons Learned from the ERPIC Mentorship Program

**DOI:** 10.5334/ijic.9844

**Published:** 2025-10-13

**Authors:** Paul Wankah, Mark Derks, Sebastian Lindblom

**Affiliations:** 1 McGill University, Canada; 2 Integrate4Health, The netherlands; 3 Karolinska Institutet, Department of Neurobiology, Care Sciences and Society, Sweden; 4 Department of Physiotherapy, School of Health, Care and Social Welfare, Mälardalen University, Västerås, Sweden

**Keywords:** integrated care, mentorship program, mentee, mentor

## Abstract

While the benefits of mentorship programs are well established, their effective design, implementation, and sustainability remain complex and challenging. This perspective paper contributes to the mentorship literature by presenting key lessons learned from the implementation of a mentorship program in the emerging field of integrated health and social care. We suggest that prioritizing thoughtful mentor-mentee matching, promoting flexible and adaptable mentoring meeting formats, offering clear guidance for structured mentoring meetings, and acknowledging the reciprocal value of mentoring relationships can inform strategic approaches to strengthening mentorship programs in integrated care and beyond.

## Introduction

Health systems globally are adopting integrated health and social care models [1,2] to improve care experiences, provider well-being, population health, and system efficiency, with notable examples including England’s Integrated Care Systems [3], Ontario Health Teams in Canada [4], and Australia’s Integrated Care Innovation Fund [5]. Organizations like the International Foundation for Integrated Care (IFIC), the King’s Fund, and the Nuffield Trust play a key role in advancing integrated care by fostering research, policy, and capacity-building through knowledge mobilization strategies such as reports, conferences, and mentorship programs.

A mentorship program is defined as a structured relationship between two people – a mentor (senior/experienced person) and a mentee (a junior/less experienced person) – in an academic, work or organizational setting where the mentor provides guidance and advice to the mentee to help them learn, build skills, and grow professionally [6,7]. Previous studies reveal that in healthcare disciplines novices (early career researchers and professionals) experience burnout due to a lack of knowledge or professional exposure [8,9,10] or feelings of frustration and depression due to unpredictable workloads [11,12] early on in their professional journey. Several disciplines have used mentorship programs to mitigate the challenges of novices in the field. For example, the Macmillan Mentorship Training Program provided 12 months mentorship to support novice nurses in their practice in the UK [13] and the Virgina Council of Nurse Practitioners-Tidewater provides leadership and mentorship opportunities to their students [14]. Some documented benefits of mentorship programs include improved satisfaction of healthcare professionals, effective support mechanism for role transitions, experiential learning and reflective practice enhance the confidence, competencies, personal and professional growth of mentees, increased retention of nurses in the field and ultimately improved patient outcomes [12,13,14,15,16].

Although mentorship programs offer numerous benefits, they also face significant challenges related to their establishment, implementation, and long-term sustainability. Challenges can arise from lack of clarity of the mentoring role that may negatively impact the mentor-mentee relationship [17], lack of mentee engagement in the mentorship program [18], excessive time and energy commitments of mentors who hold other academic and professional responsibilities [19], and ineffective mentoring pairs that hold divergent and unrealistic expectations of mentorship goals [20]. A deeper understanding of the factors that contribute to the success or failure of mentorship programs is essential to guide their effective design and implementation, particularly in the evolving field of integrated care.

## The ERPIC mentorship program

In alignment with its mission to nurture the next generation of leaders in integrated care and to support emerging researchers and professionals on advancing the science, development, knowledge and adoption of integrated care research, policy and practice, IFIC established the Early Career Researchers and Professionals in Integrated Care (ERPIC) platform in 2017 to support emerging researchers and professionals. As part of its broader capacity-building initiatives, ERPIC launched a mentorship program aimed at strengthening the skills and competencies of early-career professionals. The mentorship program was first introduced in 2018–2019 with participation from 28 mentors and 28 mentees, followed by a second cycle in 2021–2022 that included 12 mentors and 12 mentees.

The ERPIC mentorship program is structured as a one-year commitment between a mentor (senior researcher or professional associated with IFIC) and a mentee (an early career researcher or professional) with interest in the field of integrated care. ERPIC developed a toolkit of resources and information to provide basic support for the mentorship program. The toolkit outlined that the purpose of the mentoring is to support and encourage mentees to manage their learning to maximize their potential and develop skills, improving their performance in order to become the professionals they aspire to be. It also included guidance on the scope and mentoring process, a draft action plan, reflexive pre-meeting exercises, chemistry meeting and ideas for conflict resolution. Furthermore, the toolkit allows great flexibility in mentorship because it is left to the mentors and mentees to agree on the content and frequency of their meetings during the mentorship year. As part of its quality improvement efforts, ERPIC developed a mentorship survey to gather insights from both mentors and mentees, with the aim of informing future program design and implementation. This paper distills critical insights into both the strengths and challenges encountered during the implementation of the ERPIC mentorship program.

## Four key lessons learned from the ERPIC mentorship program

### Lesson 1: Effective matching of mentors and mentees is crucial for successful mentorship

Defining clear mentor-mentee matching criteria is essential to successful mentorship programs [12,21]. The first phase of the mentorship program involved pairing each mentee with a mentor. To facilitate this process, mentees were asked to submit their curriculum vitae and motivation letters, which were used to identify mentors with aligned academic and professional backgrounds. Survey feedback indicated that, overall, both mentors and mentees were satisfied with the matching process. Mentees particularly valued mentors who were active listeners, kind, knowledgeable, resourceful, and supportive of their personal and professional development. Mentors, in turn, appreciated mentees who demonstrated proactivity, enthusiasm, and clearly articulated aspirations for future achievements.

However, two key challenges related to mentor-mentee alignment emerged. First, it proved difficult to provide effective mentorship when mentees lacked a genuine interest in integrated care. Second, some highly qualified mentees primarily sought general guidance on issues such as communication strategies, scientific writing, or navigating academia, which limited the depth of mentoring in the field of integrated care. These observations underscore the importance of refining the matching process to better align mentors and mentees based on their goals, academic and professional interests, and areas of expertise. In the context of integrated care, such targeted matching is essential to fostering more meaningful, relevant and mutually beneficial mentoring relationships and experiences [14,21].

### Lesson 2: Ensuring flexibility in organizing mentoring meetings

The mentoring meeting—an intentionally scheduled and protected time for mentors and mentees to engage in discussions on the mentee’s academic and professional development—is often regarded as a cornerstone of effective mentorship programs [12,14,21]. We observed considerable variation in the frequency and duration of mentoring meetings, ranging from two to ten encounters per year, with each session typically lasting between 30 and 60 minutes. Most participants expressed a preference for virtual platforms such as Microsoft Teams and Zoom to facilitate these meetings. However, we also identified several barriers to effective engagement, including competing professional responsibilities, family commitments, and challenges related to coordinating across different time zones – particularly given the program’s global scope, which brings together participants from across multiple regions and continents. Previous studies suggest that allowing greater flexibility in scheduling or rescheduling mentoring sessions can help participants navigate these barriers, thereby enhancing participation and contributing to the overall success of the mentoring experience [22,23].

### Lesson 3: Providing guidance to structure mentoring meetings

Providing structured guidance for mentoring meetings plays a critical role in fostering successful mentorship relationships [22,23]. The ERPIC mentorship program offers a comprehensive toolkit containing practical suggestions for conducting meetings, along with a list of key discussion topics to guide meaningful mentor-mentee interactions. Feedback indicated that mentoring meetings primarily focused on two core themes: academic development and professional growth. Academic development-related discussions included theoretical considerations when studying integrated care, academic career planning, learning the mentors’ perspectives on the core concept of integrated care, exchanging academic structure, expanding the scope of thinking of the mentee on key concepts of integrated care, and scientific writing. Professional growth-related discussions included professional networking opportunities, connecting with folks working in integrated care, various career options given the training and expertise of the mentee, workplace problems and professional expectations. However, some mentors reported challenges during the mentoring meetings, noting that certain mentees showed limited interest in integrated care and professional growth —the core focus of the program— and were instead primarily seeking job opportunities. These mentors recommended that future mentees be provided with clearer guidance and structure to help align expectations and ensure more productive mentoring sessions.

Despite these challenges, mentees reported several benefits from participating in the mentorship program. Many shared that the experience helped them gain greater clarity about their professional goals and the steps needed to achieve them. Several mentees noted that the support and validation from their mentors boosted their confidence, particularly in navigating academic and professional settings. A few also indicated that the mentorship played a key role in preparing them for competitive opportunities, ultimately contributing to career advancement, such as securing postdoctoral fellowships or job placements.

### Lesson 4: Recognizing mutual benefits of mentoring relationships

Mentoring is an ongoing process that fosters professional growth, strengthens collaboration, and contributes to the long-term success of the organization. Although mentoring programs often position mentees as the primary beneficiaries, it is equally important to acknowledge the reciprocal nature of mentoring and the valuable benefits it offers to mentors as well [24,25]. Feedback from participants highlighted this mutual benefit. For instance, one mentor shared that their involvement enhanced their understanding of a different healthcare system, while another noted that it deepened their insights into the mentoring process itself. In their meta-analysis, Ghosh and Rejo [24] revealed various types of career benefits of mentoring for mentors, including improved job satisfaction, organizational commitment, job performance and career success. Taken together, recognition of mutual benefits of mentoring programs to experienced mentors may be a great driver for enhancing recruitment, participation and commitment of mentors to support mentoring programs.

## Conclusions

Mentoring programs have the potential to significantly transform the emerging field of integrated health and social care by empowering early career professionals and novice researchers to overcome challenges, build confidence, and advance in their careers. By identifying key lessons learned in the ERPIC mentorship program, we suggest practical guidance for enhancing the effectiveness, sustainability, and impact of future mentorship programs. These insights not only inform the strategic development of mentorship in integrated care but also serve as a valuable model for other emerging fields seeking to build capacity, foster professional growth, and strengthen the competencies of early-career professionals.

Building on the lessons from the ERPIC mentorship program, the next phase of integrated care mentoring should focus on strengthening mentor-mentee matching, enhancing support structures, and fostering long-term engagement. Aligning mentors and mentees by career stage and focus area can increase the relevance and impact of the experience. Providing clear guidance, practical tools, and orientation can better support participants, especially those new to mentoring. Sustaining relationships through communities of practice can extend the value of mentoring beyond the formal program. Ultimately, the ERPIC mentorship program should be positioned as a strategic lever to build leadership capacity, enable knowledge exchange, and foster global collaboration in integrated care.
